# A Review of the Relationship Between Insulin and Bone Health

**DOI:** 10.3390/biomedicines13061504

**Published:** 2025-06-19

**Authors:** Sivasree Ravindran, Sok Kuan Wong, Nur-Vaizura Mohamad, Kok-Yong Chin

**Affiliations:** 1 Department of Pharmacology, Faculty of Medicine, Universiti Kebangsaan Malaysia, Jalan Yaacob Latif, Bandar Tun Razak, Cheras, Kuala Lumpur 56000, Malaysia; p153678@siswa.ukm.edu.my (S.R.); chinky@ukm.edu.my (K.-Y.C.); 2 Centre for Drug and Herbal Development, Faculty of Pharmacy, Universiti Kebangsaan Malaysia, Jalan Raja Muda Abdul Aziz, Kuala Lumpur 50300, Malaysia; vaizura@ukm.edu.my

**Keywords:** diabetes mellitus, insulin, osteoblast, osteoclast, osteoporosis

## Abstract

Insulin, a key hormone primarily involved in glucose metabolism, has emerged as a crucial modulator of bone metabolism. Increasing evidence suggests that insulin influences bone health, but its precise mechanism of action remains unestablished. This review explores the intricate relationship between insulin and bone health, as well as elucidating the mechanism of action involved. Animal models of type 1 diabetes mellitus (T1DM) and type 2 diabetes mellitus (T2DM) demonstrated distinct skeletal alterations, largely attributed to differences in insulin availability and associated metabolic dysfunction. Insulin deficiency in T1DM was associated with the deterioration of trabecular and cortical bone, whereas insulin resistance in T2DM primarily compromised trabecular bone quality. The route, frequency, and duration of insulin administration have been shown to influence bone-related outcomes. Studies involving insulin receptor silencing have suggested that insulin signalling is essential for normal bone development and maintenance. In humans, inconsistent findings on the effects of circulating insulin levels and insulin resistance on bone health were mainly attributed to heterogeneity in age, gender, metabolic status, study designs, population characteristics, and assessment methods. This review also highlights current knowledge gaps and underscores the need for longitudinal studies and mechanistic research. A clearer understanding of the insulin–bone axis may guide the development of targeted strategies to mitigate skeletal complications in individuals with diabetes mellitus.

## 1. Introduction

Insulin is a polypeptide hormone produced by pancreatic β-cells in response to elevated blood glucose levels. It regulates glucose homeostasis by promoting glucose uptake, utilisation, and storage, while inhibiting hepatic glucose production [1]. Beyond its classical metabolic functions, insulin exerts diverse biological effects on multiple organ systems, including the cardiovascular [2], reproductive [3], central nervous [4], and skeletal systems [5]. Bone is a dynamic organ that undergoes tightly coupled remodelling, involving bone resorption by osteoclasts and bone formation by osteoblasts [6]. Osteoporosis is a leading metabolic bone disorder resulting from excessive bone resorption relative to bone formation. This imbalance causes reduced bone mass, compromised bone structural architecture, and a greater susceptibility to fractures [7].

The role of insulin in bone physiology has been extensively studied, with a complex interplay between insulin signalling and skeletal health [8,9]. Insulin supports bone formation by stimulating osteoblast proliferation and differentiation [10]. On the other hand, insulin signalling in osteoblasts promotes the osteoclasts’ ability to acidify the bone extracellular matrix, thus favouring bone resorption [9]. The global incidence of osteoporosis among patients with diabetes mellitus is approximately 28%, with those having the disease for over ten years facing a higher risk of osteoporosis. This highlights the significant impact of insulin dysregulation on bone health and the significance of osteoporosis as a comorbidity in individuals with diabetes [11]. Although numerous studies have demonstrated that insulin influences bone health, its precise mechanism of action remains unclear.

The currently available evidence reveals the important role of insulin in bone biology, but several critical research gaps remain. Animal studies vary widely in model type, insulin treatment regimens, and skeletal assessment techniques, making it challenging to compare and draw conclusions. Human studies are largely cross-sectional, with heterogeneity in population demographics and insulin resistance indices. Moreover, the effects of exogenous insulin therapy on bone health in diabetic patients are poorly defined and often confounded by disease duration, glycaemic control, and other medications. These inconsistencies underscore the need for a comprehensive synthesis of the available evidence to clarify the role of insulin in bone metabolism and identify future research priorities.

This review explores the relationship between insulin and bone health, focusing on insulin level, resistance, therapy, and the underlying mechanisms involved. By consolidating findings from both experimental and human studies, this review highlights areas of consensus, identifies conflicting results, and outlines key limitations in existing research. A clearer understanding of the connection between insulin and bone offers valuable insights for developing targeted strategies to predict, prevent, and manage skeletal complications, particularly among individuals with diabetes mellitus.

## 2. Literature Search

**Figure 1:**
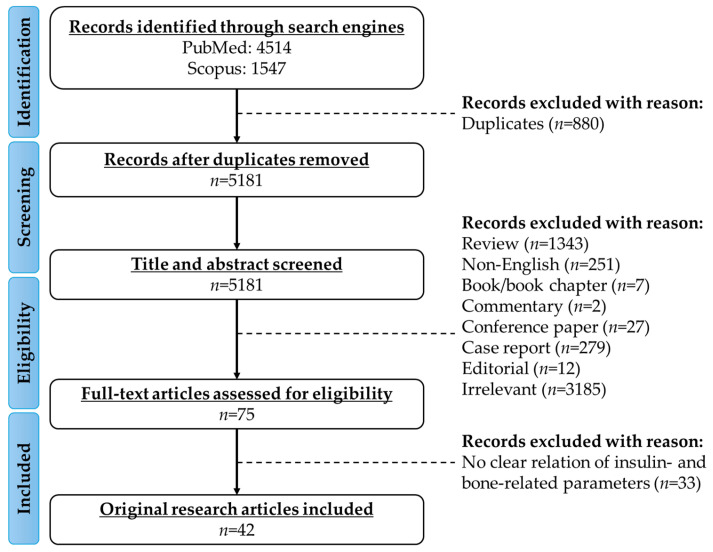
Framework of literature search.

This review aimed to explore the relationship between insulin and bone health, drawing from both animal and human studies. A comprehensive literature search was conducted using the PubMed and Scopus databases. The search employed a keyword-based approach, utilising a predefined search string: “insulin AND (level OR circulating OR treatment OR silencing OR knockdown) AND (“bone mineral density” OR “bone mineral content” bone OR osteoporosis OR osteopenia OR fracture OR osteoblast OR osteoclast OR osteocyte)”. These terms were entered directly into the databases to capture a wide range of relevant studies. The search identified 4154 records from PubMed and 1547 records from Scopus, covering the period from inception until 15 April 2025. Duplicates were removed (*n* = 880) and the remaining publications underwent thorough screening. An initial screening of titles and abstracts was conducted to remove reviews (*n* = 1343), non-English articles (*n* = 251), books/book chapters (*n* = 7), commentaries (*n* = 2), conference abstracts (*n* = 27), letters to the editor (*n* = 12), and articles unrelated to the research focus (*n* = 3185). Studies were included if they met the following criteria: (a) studies written in English; (b) animal studies that clearly described the animal model used and reported bone-related parameters such as bone mineral density (BMD), microarchitecture, or histological findings; (c) human studies that examined insulin levels, insulin resistance, or insulin treatment in relation to bone health outcomes. Data were manually extracted from the included studies, including bone-related parameters (e.g., BMD, bone formation/resorption markers), glucose and insulin-related parameters (e.g., insulin levels, insulin sensitivity/resistance, glycaemic markers), and specific bone biomarkers (e.g., osteocalcin, alkaline phosphatase). Collected references were managed using EndNote, and the extracted data were organised in Microsoft Excel for clarity and ease of analysis. Figure 1 provides an overview of the framework used for evidence collection.

## 3. Evidence from In Vivo Studies

### 3.1. Effects of Circulating Insulin Levels on Bone Health

Animal models of type 1 diabetes mellitus (T1DM) and type 2 diabetes mellitus (T2DM) displayed distinct bone phenotypes (Table 1).

Streptozotocin (STZ) injection is commonly used to induce T1DM in animal models by selectively destroying pancreatic β-cells, thereby reducing insulin levels. Hie et al. found that STZ-induced diabetic rats had reduced insulin levels, bone size, trabecular parameters, osteoblasts, and osteogenic markers, whereas their osteoclasts and bone resorption markers remained unchanged [12]. Nyman et al. reported that STZ-induced T1DM mice had higher levels of glucose, glycated haemoglobin (HbA1c), and cross-linked C-telopeptide of type I collagen (CTX), as well as reduced levels of propeptide of type 1 procollagen (P1NP), trabecular and cortical parameters, bone stiffness, force, and bending strength after eight weeks of confirmed T1DM [13].

Combining a diet high in carbohydrate, fat, and cholesterol with low-dose STZ is a widely used approach to induce T2DM in animal models as it closely mimics the metabolic characteristics of human T2DM, including insulin resistance and β-cell dysfunction. Bagi et al. found that rats fed a high-fat, high-cholesterol diet and given STZ developed hyperglycaemia and β-cell insufficiency. After 10 weeks, reduced trabecular bone measures and thinner cortical bone were seen in these rats, indicating weakened bone strength [14].

Another in vivo study by Wong et al. used a high-carbohydrate, high-fat diet to induce metabolic syndrome in rats. Metabolic syndrome is a constellation of medical conditions which is strongly associated with T2DM, primarily through insulin resistance and glucose intolerance [15]. The rats on a high-carbohydrate, high-fat diet exhibited elevated insulin, glucose, and glucose intolerance, with deteriorated trabecular bone microarchitecture and smaller bones despite unchanged cortical thickness (Ct.Th). Biomechanical tests revealed lower load and higher strain in these animals [16].

C57BL/6 mice subjected to a high-fat diet exhibited glucose intolerance and elevated fasting glucose and leptin at week 16. Bone analysis showed shorter tibial length, decreased whole-body BMD, and lower femur bone volume/total volume (BV/TV). The expressions of Runx2 and COL1 were also downregulated [17]. Using C57BL/6 mice, Sόlis-Suarez et al. observed that a hypercaloric diet combined with low-dose STZ induced hyperglycaemia, glucose intolerance, and insulin resistance. Reduced insulin levels and impaired β-cell function were linked to increased alveolar bone loss. Micro-computed tomography revealed lower alveolar bone volume and thickness, with elevated receptor activator of nuclear factor-κB ligand (RANKL) expression inversely correlated with bone mass. Diabetic mice exhibited thinner femoral cortices and decreased strength, with high glucose was associated with poorer bone density and mechanical properties [18].

Fu et al. (2015) utilised yellow Kuo Kondo (KK-Ay) diabetic mice, a classic genetic model of T2DM with a polygenic mutation characterised by elevated glucose and insulin levels, to investigate the effects of prolonged hyperinsulinaemia and hyperglycaemia on bone health [19]. High insulin levels increased cortical bone mass and density but impaired trabecular bone microstructure. Both osteogenic [OCN, bone sialoprotein (BSP), COL1, osteonectin, forkhead box protein O1 (Foxo1), Runx2, and OSX] and osteoclastogenic markers [tartrate-resistant acid phosphatase (TRAP) and V-type proton ATPase (V-ATPase)] were upregulated, indicating that insulin benefits cortical bone but adversely impacts trabecular bone [19].

The Zucker diabetic fatty (ZDF) rat model is a widely used animal model that closely mimics the metabolic characteristics of T2DM. On a high-fat, high-carbohydrate diet, these rats showed increased non-enzymatic glycation and altered bone traits, including higher cortical porosity, lower mineral density ratios, and reduced bone strength [20]. Using the same model and diet, Picke et al. induced a femoral defect in the rats. The ZDF rats showed elevated glucose, HbA1c, calcium, phosphate, CTX, and TRAP levels, and reduced P1NP levels. Bone analysis revealed lower BV/TV and bone mineral density (BMD), leading to lower biomechanical strength. Histomorphometry indicated decreased trabecular BV/TV and mineral apposition rate (MAR), with increased endocortical but decreased periosteal bone formation [21].

The Goto-Kakizaki (GK) rat model, a spontaneous T2DM model, had high insulin, glucose, TRAP, CTX, and ALP levels with reduced OCN levels. These changes resulted in lower BV/TV, mineralising surface (MS), and bone formation rate (BFR) in both trabecular and cortical bone [22]. Tsumura Suzuki Obese Diabetes mice were used to investigate the effects of T2DM on bone mass, metabolism, microstructure, and strength. The mice exhibited higher glucose and insulin levels with impaired glucose tolerance. At week 20, serum TRAP levels were elevated but bone mineral content (BMC) and BMD were lowered. Femoral microstructure changed, visualised by increased trabecular measures and decreased mid-diaphysis parameters at week 20. Histological analysis revealed thicker cortical bone in diabetic mice [23]. In Nagoya Shibata Yasuda (NSY) mice, rising glucose with stable insulin impaired glucose tolerance. Serum OCN and TRAP declined, correlating with hyperglycaemia. Femoral BMD, BMC, and mechanical strength were deteriorated at week 20 [24].

In brief, animal models of T1DM and T2DM exhibit distinct bone phenotypes primarily due to differences in insulin levels, glucose metabolism, and disease progression. T1DM models, typically induced by STZ, demonstrate pronounced reductions in circulating insulin along with marked deterioration in both trabecular and cortical bone quality. Interestingly, the onset period of T1DM in animals also influences outcomes, as early onset has less effect on bone, particularly the cortical part, compared to the long-term effects of T1DM. The time of onset seems to significantly affect bone markers, as a decrease in the bone absorption marker and increase in bone resorption marker were observed in the early-onset stage [13]. On the other hand, T2DM models (induced through a combination of STZ and diets rich in fats, carbohydrates, and cholesterol, or via genetic modifications) display impaired trabecular architecture with comparatively minimal cortical bone thinning. It was also noticeable that the bone resorption markers, especially TRAP levels, were mostly elevated in animals that displayed compromised trabecular bone and unaffected or minimal adverse effects in cortical bone. These findings highlight the complex and differential effects of diabetic conditions on skeletal health, emphasising the importance of developing tailored strategies for managing skeletal complications in the two different types of diabetes.

**Table 1:** The relationship between circulating insulin levels on bone health in animal studies.

Type of Animal Model	Findings	Reference
Rats injected with STZ	Insulin: ↓; insulin receptor: ↔; femur length: ↓; tibia length: ↓; bone weight (femur, tibia, proximal tibia): ↓; ALP: ↓; TRAP: ↔; CTSK: ↔; calcium: ↓; hydroxyproline: ↓; deoxypyridinoline: ↔; BV/TV: ↓; trabecular bone surface: ↓; Tb.Th: ↓; Tb.N: ↓; Ob.N: ↓; Oc.N: ↔; BMP-2: ↔; Dlx5: ↓; Runx2: ↓; OSX: ↓; OCN: ↓; COL1: ↓; Wnt3a: ↔; LRP5: ↔; Akt: ↔; p-Akt: ↓; GSK3β: ↔; p-GSK3β: ↓; β-catenin: ↓; p-β-catenin: ↓; Sost: ↑; DKK1: ↑	[12]
Mice injected with STZ	Glucose: ↑; CTX: ↑; P1NP: ↓; stiffness: ↓; force: ↓; femoral length: ↔; modulus: ↔; bending strength: ↓; toughness: ↔ Cortical parameters: Ct.Ar: ↓; Ct.Th: ↓; mineral density: ↔ Trabecular parameters: BV/TV: ↓; Tb.N: ↓; Tb.Th: ↓; mineral density: ↓	[13]
Rats injected with STZ and fed high trans-fat diet with 2% cholesterol	Insufficiency of β-cells; glucose: ↑; insulin: ↔; tibial length: ↔; tibial width: ↔; stiffness: ↔; load: ↔; strength: ↓ Cortical parameters: BMC: ↔; BMD: ↔; Ct.Th: ↓; periosteal perimeter: ↔; endosteal perimeter: ↑ Trabecular parameters: BMD: ↔; BV/TV: ↓; bone surface: ↓; Tb.N: ↓; Tb.Th: ↔; Tb.Sp: ↑; SMI: ↔; Conn.D: ↓	[14]
Rats with metabolic syndrome induced by high-carbohydrate high-fat diet	Insulin: ↑; glucose: ↑; calcium: ↔; load: ↓; displacement: ↔; stiffness: ↔; stress: ↔; strain: ↑; elasticity: ↔; Ob.S: ↓; Oc.S: ↔; ES: ↑; OS: ↓; OV: ↓; sLS: ↔; dLS: ↔; MS: ↔; MAR: ↔; BFR: ↔ Cortical parameters: Ct.Th: ↔; Ct.Ar: ↔; Tt.Ar: ↓ Trabecular parameters: BV/TV: ↓; Tb.N: ↓; Tb.Sp: ↑; Tb.Th: ↔; SMI: ↑; Conn.D: ↓	[16]
C57BL/6 mice fed high-fat diet	Fasting glucose: ↑; fasting insulin: ↑; glucose intolerance: ↑; leptin: ↑; tibia length: ↓; whole body BMD: ↓; Runx2: ↓; COL1: ↓ Cortical parameters: Ct.Th: ↔; Ct.Ar: ↔ Trabecular parameters: BV/TV: ↓	[17]
C57BL/6 mice injected with low-dose STZ and fed hypercaloric diet	Glucose: ↑; insulin: ↓; HOMA-β cell function: ↓; alveolar bone loss: ↑; alveolar RANKL: ↑; load: ↓; force: ↓; stiffness: ↓; elastic modulus: ↓; stress: ↓ Cortical parameter: Ct.Th: ↓ Trabecular parameter: BV/TV: ↓; Tb.Th: ↓; Tb.Sp: ↑	[18]
KK-Ay diabetic mice	Insulin: ↑; glucose: ↑; OCN: ↑; BSP: ↑; COL1: ↑; osteonectin: ↑; ALP: ↓; Foxo1: ↑; Runx2: ↑; OSX: ↑; TRAP: ↑; V-ATPase: ↑ Cortical parameters: BMD: ↑; Ct.Th: ↑ Trabecular parameters: BMD: ↓; BV/TV: ↓; Tb.Th: ↓; Tb.N: ↔; Tb.Sp: ↑	[19]
Zucker diabetic fatty rats fed high-fat, high-carbohydrate diet	Non-enzymatic glycation: ↑; mineral density ratio: ↓; elastic modulus: ↔; toughness: ↓; maximum stress: ↓ Cortical parameters: Porosity: ↑	[20]
Zucker diabetic fatty rats with subcritical femur defects	Glucose: ↑; HbA1c: ↑; calcium: ↑; phosphate: ↑; P1NP: ↓; CTX: ↑; TRAP: ↑; PTH: ↔; force: ↓; work to failure: ↓; BFR: ↓; MS: ↓; MAR: ↓ Cortical parameters: BV/TV: ↓; BMD: ↓; Tb.N: ↓; Tb.Th: ↓ Trabecular parameters: BV/TV: ↓; BMD: ↓; Ct.Th: ↓	[21]
Goto-Kakizaki rats	Insulin: ↑; OCN: ↓; ALP: ↑; CTX: ↑; TRAP: ↓; calcium: ↔; phosphate: ↓; BMD: ↓; Ob.S: ↓; Oc.S: ↔; MS: ↓; MAR: ↓; BFR: ↓; load: ↓; stiffness: ↓ Cortical parameters: Ct.Ar: ↓ Trabecular parameters: BV/TV: ↓; Tb.N: ↓; Tb.Th: ↓; Tb.Sp: ↑; Conn.D: ↓	[22]
Tsumura Suzuki Obese Diabetes mice	Glucose: ↑; insulin: ↑; glucose intolerance: ↑; OCN: ↔; TRAP: ↑; BMC: ↓; BMD: ↓; force: ↔; stiffness: ↔; work to failure: ↔; stress: ↓; elastic modulus: ↔; toughness: ↔ Cortical parameters: Ct.V: ↔; Tt.V: ↓; Ct.V/Tt.V: ↑; porosity: ↔; Ct.Th: ↑ Trabecular parameters: BV/TV: ↑; Tb.Th: ↑; Tb.N: ↑; Tb.Sp: ↓	[23]
Nagoya Shibata Yasuda mice	Glucose: ↑; insulin: ↔; glucose intolerance: ↑; femur length: ↓; BMD: ↓; BMC: ↓; OCN: ↓; TRAP: ↓; force: ↓; stiffness: ↔; work to failure: ↔; stress: ↔; elastic modulus: ↓; toughness: ↓ Cortical parameters: Ct.Ar: ↓; Ct.Th: ↔ Trabecular parameters: Tb.Ar: ↔	[24]

Abbreviations: Akt, protein kinase B; ALP, alkaline phosphatase; BFR, bone formation rate; BMC, bone mineral content; BMD, bone mineral density; BMP-2, bone morphogenic protein-2; BSP, bone sialoprotein; BV/TV, bone volume/total volume; COL1, type 1 collagen; Conn.D, connectivity diameter; CTSK, cathepsin K; Ct.Ar, cortical area; Ct.Th, cortical thickness; Ct.V, cortical volume; CTX, carboxyl-terminal cross-linked telopeptide of type 1 collagen; DKK1, Dickkopf-related protein 1; dLS, double-labelled surface; Dlx5, distal-less homeobox 5; ES, eroded surface; Foxo1, forkhead box protein O1; GSK3β, glycogen synthase kinase 3-beta; HbA1c, glycated haemoglobin A1c; PTH, intact parathyroid hormone; KK-Ay, yellow Kuo Kondo; LRP5; lipoprotein receptor-related protein 5; MAR, mineral apposition rate; MS, mineralising surface; Ob.N, osteoblast number; Ob.S, osteoblast surface; Oc.N, osteoclast number; Oc.S, osteoclast surface; OCN, osteocalcin; OS, osteoid surface; OSX, osterix; OV, osteoid volume; P1NP, N-terminal propeptide of type 1 collagen; p-Akt, phosphorylated protein kinase B; p-β-catenin, phosphorylated β-catenin; p-GSK3β, phosphorylated glycogen synthase kinase 3-beta; RANKL, receptor activator of nuclear factor kappa-B ligand; Runx2, runt-related transcription factor 2; sLS, single-labelled surface; SMI, structural model index; Sost, sclerotin; Tb.N, trabecular number; Tb.Sp, trabecular separation; Tb.Th, trabecular thickness; TRAP, tartrate-resistant acid phosphatase; Tt.Ar, total cross-sectional area inside periosteal envelope; Tt.V, total bone volume; V-ATPase, V-type proton ATPase; Wnt3a, Wnt ligand 3a; β-catenin, beta-catenin; ↑, increase; ↓, decrease; ↔, no change.

### 3.2. Effects of Insulin Treatment on Bone Health

Animal studies suggested that insulin treatment exerted positive effects on bone health in both T1DM and T2DM model rats (Table 2). One study showed that subcutaneous insulin injections had anabolic effects on bone, evidenced by improved trabecular bone microstructure, higher serum calcium, osteogenic marker (OCN), and anti-osteoclastogenic cytokine (osteoprotegerin, OPG) levels in STZ-induced diabetic animals as compared to the nontreated group [25]. In another study, STZ-induced diabetic rats treated with insulin via subcutaneous injection experienced lesser alveolar bone loss compared to non-diabetic controls, with higher OPG levels and unchanged RANKL levels [26].

Insulin administration via osmotic minipump in STZ-induced diabetic mice showed negative linear relationships with glucose levels and CTX, and a positive linear relationship with P1NP expression. Femoral midshaft analysis revealed increases in cortical area (Ct.Ar), Ct.Th, stiffness, and peak force in response to insulin administration [13]. Similarly, subcutaneous insulin infusion also conferred bone protection in STZ-induced diabetic rats. The diabetic rats had a higher bone size, denser bone microarchitecture, and increased osteogenesis-related markers after 4 weeks of treatment [12].

Using Zucker diabetic fatty rats on a high-fat, high-carbohydrate diet as an animal model for T2DM, subcutaneous insulin injection (0.5 IU to 13.0 IU over 12 weeks) did not change cortical porosity, bone tissue mineral density, or the bone mechanics (stress and toughness) of the femoral midshaft when compared to untreated diabetic controls [20]. In another study using Zucker diabetic fatty rats with subcritical femur defects, subcutaneous insulin injection (0.5 IU to 13.0 IU over 12 weeks) reduced glucose and HbA1c levels. Cortical bone mass was increased but trabecular bone microstructure remained unchanged. The bone formation rate and P1NP and TRAP levels increased, indicating enhanced bone turnover [21]. Conversely, continuous subcutaneous insulin infusion (2.5 IU/day for four weeks) improved trabecular bone microstructure and increased bone toughness. Insulin treatment upregulated OCN and OPG expression while downregulating RANKL, indicating enhanced osteogenesis and suppressed osteoclastogenesis [27].

These animal experimental studies highlighted several key findings. Insulin plays a dual role in bone health: it promotes bone formation while inhibiting bone resorption [13]. Twice-daily subcutaneous administration of insulin (2.5 IU) for eight weeks was insufficient to increase ALP activity and alter the expression of RANKL [25]. Meanwhile, continuous subcutaneous infusion of insulin (1.6 IU) for four weeks increased ALP expression in the proximal tibia [12]. In addition, RANKL expression in the trabecular and cortical regions was downregulated after continuous subcutaneous infusion of insulin (2.5 IU) for four weeks [27]. Specifically, the reduction in RANKL can be offset by an increase in OPG, which supports enhanced bone formation in both T1DM and T2DM [25,26,27]. Thus, the bone-protective effects of insulin treatment might be influenced by treatment frequency. The ALP measurement site also varied between studies. An extended period of insulin infusion (eight weeks) provided greater bone protection compared to a four-week duration. An eight-week continuous subcutaneous insulin infusion of (2.5 IU/day) also enhanced bone strength, including load, elasticity, and hardness, while reducing TRAP expression, which was not seen in four-week insulin infusion [27].

### 3.3. Effects of Insulin Receptor Silencing on Bone Health

Knockout animal models have been used to investigate the impact of insulin receptor deficiency on bone homeostasis (Table 3). A study by Studentsova et al. indicated that the deletion of insulin receptor β (IRβ) in mice did not cause any alteration in either trabecular or cortical bone microstructure as compared to wild-type controls at 15 weeks of age. A dramatic impairment in trabecular bone microstructure was noted in mice with IRβ deletion when compared to the wild-type controls at 48 weeks of age. Significant reductions in trabecular BV/TV and torsional rigidity were detected in the femurs. However, no significant change was observed in the cortical bone microstructure at week 48 between the mice with IRβ deletion and wild-type controls [28]. In another study, mice with insulin receptor deficiency in osteoblasts showed decreased BV/TV, Tb.N, Tb.Th, Ob.N, and erosion depth, along with increased Tb.Sp and OPG, while their osteoclast number (Oc.N) remained unchanged as compared to controls. Consistent with this, the circulating OCN level was lowered in these mice, indicating reduced bone formation. These findings suggest that insulin receptors play a crucial role in normal bone acquisition, and the observed deterioration in bone phenotypes can likely be attributed to impaired osteoblast maturation [29].

Thrailkill et al. conducted a study using male and female mice with osteoprogenitor-selective ablation of their insulin receptors to examine the role of insulin receptors in bone development. This model represents a model of early insulin receptor elimination in osteoprogenitor cells during osteoblast development. The femurs of these mice were slender, shorter, and exhibited reduced cortical bone structure (Ct.Th, Ct.Ar, and mineral density) compared to the control mice. However, the skeletal changes displayed gender-specific differences despite their overall size reduction. In male mice, the loss of insulin receptors in osteoprogenitors resulted in the significant deterioration of the trabecular bone microstructure, as evidenced by reduced BV/TV and Tb.Th and increased rod-like trabeculae in the femoral metaphysis. In contrast, female mice showed largely unaffected trabecular bone architecture, with a lower structural model index (SMI), suggesting fewer rod-like trabeculae. The authors postulated that this preservation of trabecular bone microstructure in female mice may be due to the protective effects of oestrogen on metaphyseal bone [30].

Transgenic insulin receptor knockout mouse models serve as excellent animal models to examine the role of reduced insulin signalling in T1DM while maintaining normal glucose levels. An earlier study by Irwin et al. found that transgenic insulin receptor knockout mice exhibited a higher cortical bone area/total area (BA/TA) and a lower medullary area than wild-type controls. However, no significant differences were observed in tibial length or trabecular bone parameters as compared to the control group. Additionally, serum pyridinoline level (a bone resorption marker) and tibial expressions of Runx2 and OCN were comparable between both groups [31]. The findings of this study suggested that the lack of insulin signalling without hyperglycaemia in mice did not adversely affect bone density.

Several key points can be concluded based on these findings. The deletion of insulin receptors at the early stage of osteoblast differentiation in osteoprogenitors resulted in structural, architectural and biomechanical deterioration in cortical bone. At the trabecular region, male mice experienced a greater decline in bone parameters than female mice. This difference might be due to the protective role of oestrogen, which directly inhibits bone resorption. Meanwhile, the beneficial effects of testosterone on bone health are primarily mediated through its conversion to oestrogen within the body. Furthermore, the absence of insulin receptors in mature osteoblasts primarily affects trabecular rather than cortical bone and increases the susceptibility of bones to age-related deterioration. Bone-related changes might take longer to manifest following the loss of insulin signalling through receptor silencing or deletion in mature osteoblasts. Collectively, the available evidence suggests that insulin receptor expression in osteoprogenitors and osteoblasts is important for bone development and maintenance. Further investigation is needed to understand the underlying mechanisms of these effects better.

## 4. Evidence from Human Studies

### 4.1. Effects of Circulating Insulin Levels on Bone Health

Earlier studies on the relationship between serum insulin level and bone health revealed conflicting outcomes showing beneficial, detrimental, and negligible effects (Table 4). Two cross-sectional studies revealed a positive relationship between serum insulin level and BMD in non-diabetic postmenopausal women [32,33]. In the earlier study, the postmenopausal women in the higher quartile of insulin level had higher hip BMD and volumetric BMD in the trabecular, cortical, and integral (trabecular and cortical) compartments [32]. A subsequent study by Ye et al. pointed out that postmenopausal women with higher fasting insulin levels had higher femoral neck BMD as compared to those with lower fasting insulin levels [33]. In contrast, a high circulating insulin level might not be beneficial in adolescents. A larger cross-sectional study involving 2784 boys and girls with a mean age of 15.5 years old reported distinct outcomes. Peripheral quantitative computed tomography analysis showed a negative association between plasma insulin level and periosteal circumference at the mid-tibia. Insulin also demonstrated an inverse relationship with cortical BMD [34]. The results of this study were solely based on measurements taken at a single site (mid-tibia), which might have contributed to the observed discrepancy. In a sample of older adults comprising men and women aged 56.8 ± 11.3 years old (*n* = 717), higher fasting insulin was associated with lower femoral neck BMD, lumbar spine BMD, compression, bending, and impact strength index values [35]. In a cross-sectional study involving 466 young adults (male: 19.8 ± 2.3 years old; female: 19.4 ± 2.2 years old), there was no association between insulin levels and total body BMC after adjusting for age, sex, and total lean mass [36].

In short, serum insulin level affects bone health differently across age groups and populations. A higher insulin level was associated with reduced bone quality in adolescents [34], possibly attributed to ongoing bone growth meaning that peak bone mass has not yet been achieved. In young adults, no significant association has been found between insulin level and BMC [36], likely due to the optimal insulin sensitivity and well-regulated insulin levels in this age group. In older adults, an elevated fasting insulin level was correlated with lower BMD along with decreased bone strength [35]. Insulin resistance becomes more prevalent with ageing, contributing to chronic hyperinsulinaemia. Persistent hyperinsulinaemia induces a pro-inflammatory state, oxidative stress, and increased bone marrow adiposity, thereby impairing osteoblastogenesis and promoting bone resorption [37,38]. In postmenopausal women, a higher insulin level was correlated with increased BMD primarily due to higher BMI [32,33]. The heterogeneous outcomes underscore the need for longitudinal studies to establish causality rather than relying on cross-sectional data. Further investigation into insulin resistance may provide a deeper understanding of the role of insulin in bone health.

### 4.2. Effects of Insulin Resistance on Bone Health

Insulin resistance is a major risk factor for developing T2DM, hallmarked by the impaired response of the body to insulin, resulting in elevated blood sugar levels. Several indices are used to provide insight into insulin resistance, insulin sensitivity, and β-cell function using fasting glucose, fasting insulin, lipids, and anthropometric measures. Homeostatic model assessment of insulin resistance (HOMA-IR) and homeostatic model assessment of β-cell function (HOMA-β) are two traditional markers. HOMA-IR is a method for estimating insulin resistance (calculated using fasting glucose and fasting insulin levels), with a higher value indicating greater insulin resistance [39]. HOMA-β is another index derived from fasting glucose and fasting insulin levels, which evaluates pancreatic β-cell insulin secretory function. A lower HOMA-β value suggests β-cell dysfunction, commonly observed in type 2 diabetes [40]. The quantitative insulin sensitivity check index (QUICKI) offers a refined alternative for estimating insulin sensitivity based on fasting glucose and fasting insulin levels, whereby a higher value suggests greater insulin sensitivity [41]. The triglyceride–glucose index (TyG) measures insulin resistance using fasting triglyceride and fasting glucose levels. Its variants [including TyG index with waist circumference (TyG-WC), TyG index with waist-to-height ratio (TyG-WHtR), and TyG index with body mass index (TyG-BMI)] integrate anthropometric data as additional factors to improve its accuracy in assessing insulin resistance. A higher TyG index is associated with increased insulin resistance. They are useful markers for obesity-related insulin resistance [42]. On the other hand, the metabolic score for insulin resistance (METS-IR) is a novel non-insulin index (calculated from fasting blood glucose, triglyceride, high-density lipoprotein cholesterol and BMI) that evaluates insulin resistance, providing a comprehensive overview of insulin resistance in metabolic disorders [43]. A wide array of scientific evidence has revealed the relationship between insulin resistance and bone health in humans with heterogeneous findings (Table 5).

In boys and girls aged 13.93 ± 2.64 years old (*n* = 423), HOMA-IR showed an inverse relationship with most of the bone parameters [including BMC, BMD, bone mineral apparent density (BMAD), and Z-score] at the lumbar spine, femoral neck, and whole body. A significant positive correlation between QUICKI and bone parameters for the lumbar spine and femoral neck was also detected [44]. In a study using publicly available data from the National Health and Nutrition Examination Survey (NHANES) comprising 5292 participants aged 18 years old and above, it was found that participants with HOMA-IR ≥ 2 and HOMA-β < 100 had an increased risk of osteoporosis in contrast to those with HOMA-IR < 2 and HOMA-β < 100. Additionally, a greater risk of osteoporosis at the femoral neck was detected in participants who had HOMA-IR ≥ 2 and HOMA-β ≥ 100, compared to those with HOMA-IR < 2 and HOMA-β < 100 [45]. Zhuo et al. also found detrimental effects of insulin resistance on bone, whereby the TyG index was found to have an association with the likelihood of low bone mass and osteoporosis in men and women [46]. Srikanthan et al. observed that greater HOMA-IR values were correlated with lower femoral neck strength and lumbar spine BMD in men and women (*n* = 717; aged 56.8 ± 11.3 years) recruited in the biomarker project of the Midlife in the United States Study [35].

Low bone turnover markers were unique to insulin resistance and T2DM. In a cross-sectional analysis of the Dubbo Osteoporosis Epidemiology Study, 525 men and women aged 60 years old and above were recruited to characterise the relative association of insulin resistance and T2DM with bone turnover markers. A multivariable analysis found that insulin resistance and T2DM were associated with low CTX levels in men. Similarly, a similar association was noted for the levels of OCN, P1NP, and CTX in women with T2DM [47]. Likewise, the levels of CTX, P1NP, and OCN decreased as HOMA-IR quartiles increased in another larger cross-sectional study conducted among populations with diabetes and hyperglycaemia (*n* = 5277; aged ≥ 18 years old) in China. Additionally, HOMA-β showed a positive relationship with the three bone turnover markers [48]. Collectively, the findings of these two studies indicated that higher insulin resistance and lower β-cell function were related to impaired bone remodelling.

Several studies examined the relationship between insulin resistance and bone health in midlife women. A study by Shieh et al. uncovered that lower insulin resistance preserved BMD by slowing its decline, whereas higher insulin resistance was associated with rapid BMD decline in premenopausal, menopausal transition, and postmenopausal women [49]. In postmenopausal Korean women (*n* = 1008; aged ≥ 50 years old), a higher HOMA-IR value was correlated with reduced total bone volume, cortical volume, and femoral neck strength. However, cortical BMD was higher in postmenopausal women with higher HOMA-IR values, which could be explained by their higher body weight and mechanical loading [50]. In community-dwelling overweight and obese adults aged 62.8 ± 7.9 years (36 men and 43 women), HOMA-IR was negatively linked to the cortical density of the proximal radius in women. At the same time, no significant association was found between HOMA-IR and any bone parameters in men [51]. In patients with T2DM aged 57.5 ± 10.8 years old (*n* = 234), an increase in HOMA-IR elevated the risk of osteoporosis in women [52]. These cross-sectional studies involved relatively small sample sizes, limiting the generalisability of their findings to the broader older population.

Contrary to the aforementioned studies, some studies reported insulin resistance as a protective factor for bone health. In younger adults (*n* = 5456; aged 30.33 ± 13.55 years old), a positive relationship between total BMD and TyG, TyG-WC, TyG-WHtR, and TyG-BMI was identified [53]. In postmenopausal women, a cohort study by Cherif et al. revealed that BMD at the left femur and total hip was higher in individuals with insulin resistance compared to those without it [54]. Ye et al. recruited 437 non-diabetic postmenopausal women aged 51–56 years old in a cross-sectional study. Femoral neck BMD and T-score were higher in non-diabetic postmenopausal women with higher levels of HOMA-IR, HOMA-β, and METS-IR [33]. The results obtained by Campillo-Sánchez et al. in a cross-sectional study involving 381 non-diabetic postmenopausal women with suspected or diagnosed osteoporosis pointed out higher hip and volumetric BMD in women with higher HOMA-IR values. However, a similar trend was not seen for trabecular bone score in non-diabetic postmenopausal women [32]. In a retrospective study involving postmenopausal T2DM patients, METS-IR scores were lower in the osteoporotic group than in the non-osteoporotic group. A positive correlation was detected between METS-IR scores and lumbar vertebrae, femoral neck, and hip BMD. These findings reiterated that METS-IR was a protective factor for osteoporosis in postmenopausal women with T2DM [55].

Taken together, the current evidence reveals a complex relationship between insulin resistance and bone health. Various indices such as HOMA-IR, HOMA-β, QUICKI, TyG, and MET-IR can be used to offer insights into insulin resistance/sensitivity and pancreatic β-cell function in humans. Increased insulin resistance was associated with lower BMD, increased risk of osteoporosis, and reduced bone turnover markers and bone strength, evident across different populations, including adolescents, young to older adults, premenopausal to postmenopausal women, overweight to obese subjects, and T2DM patients [35,44,45,46,47,48,49,50,51,52]. Some studies have reported that the negative impacts of insulin resistance on bone health were seen in women but not in men [47,51]. Gender may influence the impact of insulin resistance on bone health due to changes in hormonal levels, with women being more susceptible than men, particularly at the postmenopausal stage.

The protective impact of insulin resistance has been reported in younger adults and postmenopausal women, whereby it was correlated with higher BMD [32,33,53,54,55]. It has been postulated that insulin resistance is associated with higher body weight, which exerts greater mechanical stress on bone cells to stimulate osteoblast activity and promote bone formation [56]. Compensatory hyperinsulinaemia occurs in the state of insulin resistance. Insulin has anabolic effects on osteoblasts, thus promoting osteoblast proliferation, bone formation, and mineralisation [8,19]. In addition, variations in study design (such as cross-sectional or longitudinal), population characteristics, and measurement techniques may contribute to inconsistencies in the reported outcomes.

The limitations of the current evidence need to be acknowledged. Firstly, most of these studies were conducted with a cross-sectional design, thus limiting the possible inferences on causal and temporal relationships. Longitudinal studies will be helpful to clarify whether insulin resistance is causally and temporally linked to impaired bone health. Secondly, some studies were confined to specific communities, making their findings less applicable to individuals from different regions and ethnic backgrounds. Thirdly, only a paucity of studies investigated the effects of insulin resistance on biochemical markers of bone formation and resorption. Further research can be improved by incorporating a more comprehensive assessment of bone density, microarchitecture, biochemical markers, bone strength, fracture risk, and events at various skeletal sites. Additionally, utilising various insulin resistance indices can provide deeper insights into whether insulin resistance deleteriously affects bone health.

### 4.3. Effects of Insulin Treatment on Bone Health

Insulin therapy is used to treat insulin insufficiency or resistance among patients with diabetes mellitus. Insulin treatment has shown diverse effects on bone health, with studies suggesting protective benefits, negative impacts, and no significant effects (Table 6).

Roomi et al. conducted a cohort study by recruiting 200 subjects aged 50–73 years old. The participants were divided into four groups, including healthy postmenopausal women, postmenopausal women with osteoporosis, T2DM postmenopausal women treated with insulin once daily, and T2DM postmenopausal women treated with metformin (500 mg) twice daily. The subjects were treated with insulin or metformin for three years. The authors found that the BMD was higher, but bone markers (OCN and CTX) were lower in postmenopausal women with T2DM and treated with insulin as compared to postmenopausal women with osteoporosis [57]. In a retrospective study, Liu et al. included a total of 80 T2DM patients aged 30 to 60 years old receiving oral glucose-lowering medication or insulin injections for more than one year. The T2DM disease duration of the patients was 5.91 ± 3.39 years. T2DM patients who received both oral glucose-lowering medication and insulin glargine injection had higher BMD at the lumbar vertebrae and spine than those who took oral glucose-lowering medication only. Higher calcium and lower phosphate levels were also recorded in patients who received both oral glucose-lowering medication and insulin glargine [58].

The study by Ivaska et al. investigated the changes in bone formation and resorption markers in response to acute hyperinsulinaemia in men and women from three independent cohorts (normal adults, young men, and older women). In normal adults (aged 37–63 years old), the bone resorption marker (CTX) was reduced in response to four hours of low-dose insulin infusion. In young men (aged 18–34 years old), high-dose insulin infusion for four hours reduced uncarboxylated osteocalcin (ucOCN) and CTX levels. For elderly women (aged 69–79 years old), two hours of low-dose insulin infusion decreased TRAP, ucOCN, and total OCN levels. However, no changes were found in the circulating levels of P1NP in all the cohorts [59]. This study concluded that insulin infusion decreased bone resorption markers but to a lesser extent for bone formation markers. However, direct comparison between the three independent cohorts was limited due to differences in baseline characteristics as well as variations in insulin infusion rates and durations.

On the contrary, a study by Ruppert et al. revealed that women with T2DM who used insulin (*n* = 55; aged 53.9 ± 5.7 years old) experienced a greater reduction in femoral neck BMD as compared to those who did not use insulin (*n* = 55; aged 53.3 ± 4.9 years old). The duration of diabetes was 9.1 years in the insulin user group and 5.7 years in the non-insulin user group, indicating a more advanced disease state in those using insulin. Therefore, BMD was reduced with the prolongation of T2DM duration [60]. In a large population-based study in Spain, the results found that insulin use in the early stages of T2DM was associated with a 38% increased risk of fractures as compared to non-insulin user [61].

Study by Basu et al. reported a negligible effect of insulin therapy on bone whereby insulin infusion (at low, intermediate, or high doses) did not influence the levels of bone markers such as P1NP, OCN, ucOCN, CTX, and OPG in diabetic and non-diabetic subjects (*n* = 14; aged 59.9 ± 2.2 years old), most likely due to the relatively small sample size in this cohort [62].

In brief, the effects of insulin therapy on bone health remain inconclusive, with mixed results likely influenced by disease stage and treatment duration. It is well established that bone quality deteriorates as the duration of T2DM increases. Consequently, individuals in the advanced stage of the disease may experience low BMD despite receiving insulin treatment. A study has reported that recently diagnosed type 2 diabetes mellitus requiring insulin therapy was associated with an increased risk of major fractures within five years [61]. Hence, insulin treatment for less than five years may have beneficial effects on bone [57,58]. Also, the type of insulin used, in matters of at meal times or long-acting insulin administered at night, did not prove to affect bone formation and resorption processes, as evidenced by the respective markers [63].

## 5. The Mechanistic Pathway of Insulin

### 5.1. Phosphatidylinositol 3-Kinase (PI3K)/Protein Kinase B (Akt)/Glycogen Synthase Kinase 3-Beta (GSK3β) Pathway

**Figure 2:**
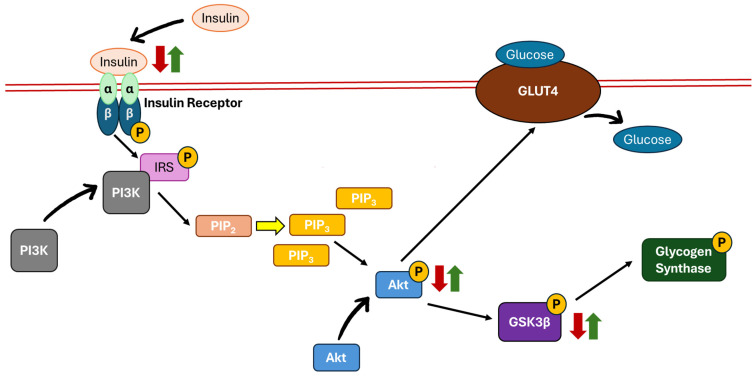
The PI3K/Akt/GSK3β pathway plays a crucial role in insulin signalling. A reduced insulin level suppresses the activation of the PI3K/Akt/GSK3β pathway (indicated by red arrows), leading to downstream effects such as decreased glucose uptake and glycogen synthesis. Conversely, insulin binding to its receptor enhances PI3K/Akt/GSK3β activation, promoting glucose uptake and glycogen synthesis (indicated by green arrows). Abbreviations: Akt, protein kinase B; GLUT4, glucose transporter 4; GSK3β, glycogen synthase kinase 3-beta; IRS; insulin receptor substrate; PI3K, phosphoinositide 3-kinase; PIP_2_, phosphatidylinositol 4,5-bisphosphate; PIP_3_, phosphatidylinositol 3,4,5-trisphosphate; P, phosphorylation; ↑, increase; ↓, decrease.

The β-cells in the pancreatic islets secrete insulin in response to fluctuations in glucose, amino acid, and free fatty acid levels. Insulin is crucial in maintaining glucose regulation, primarily through glucose uptake via the translocation of glucose transporter 4 (GLUT4) into muscle and fat cells [64]. The regulation of glucose level modulated through the PI3K/Akt pathway begins with the binding of insulin to its receptor. The insulin receptor consists of two alpha (α) units and two beta (β) units. When insulin binds to the α units, it triggers the auto-phosphorylation of the β units, activating insulin receptor substrate (IRS). This activation recruits PI3K to subsequently phosphorylates phosphatidylinositol 4,5-bisphosphate (PIP2) to form phosphatidylinositol 3,4,5-trisphosphate (PIP3). Next, Akt is recruited and activated as the level of PIP3 increases, leading to the aggregation of GLUT4 on the cell membrane and enhancing glucose uptake. On the other hand, Akt also inhibits GSK3β. In its active state, GSK3β inhibits glycogen synthase, which negatively affects glucose uptake. When GSK3β is inactivated, it leads to the phosphorylation of glycogen synthase, promoting glycogen synthesis and contributing to overall glucose homeostasis [37,65]. A study by Hie et al. demonstrated lower insulin leading to the lower phosphorylation of Akt and GSK3β in STZ-induced diabetic rats. These changes were reversed by the continuous subcutaneous infusion of insulin (1.6 IU/day) for four weeks [12] (Figure 2).

### 5.2. Wnt/β-Catenin Pathway

The canonical Wnt/β-catenin pathway is essential for regulating cell proliferation, differentiation, apoptosis, migration, invasion, and tissue homeostasis [45,66]. Its activation also promotes bone formation by modulating osteoblast activity [67]. The Wnt signalling pathway is initiated when Wnt ligands bind to their receptors [including Frizzled and low-density lipoprotein receptor-related protein 5 (LRP5)] on the cell surface, triggering downstream signalling cascades. Upon ligand binding, the protein dishevelled (DVL) is activated, facilitating the aggregation of the destruction complex. Simultaneously, the ligand binding causes axis inhibition protein (AXIN) to interact with LRP5. As a result, GSK3β is phosphorylated, leading to its inhibition. This leads to the release of unphosphorylated β-catenin from the destruction complex, allowing its accumulation in the cytoplasm. Subsequently, β-catenin translocates into the nucleus, where it interacts with T-cell-specific factor (TCF) and lymphoid enhancer-binding factor (LEF). This interaction promotes the transcription of bone-forming genes and regulates the differentiation of pre-osteoblasts by the induction of Runx2 and OSX [45,66,67].

The inactivation of the Wnt signalling pathway primarily occurs through inhibitors such as Dickkopf-related protein 1 (DKK1) and sclerostin (Sost) produced by osteoblasts [68], which target LRP5 as well as secreted Frizzled-related proteins (sFRPs), which inhibit the Frizzled receptor. These inhibitors block Wnt ligand binding, thereby preventing downstream signalling. In the absence of Wnt ligand binding to its receptors, a destruction complex is formed, consisting of AXIN, adenomatous polyposis coli (APC), GSK3β, and casein kinase alpha (CK1α). CK1α and GSK3β then phosphorylate β-catenin, leading to its ubiquitination and subsequent degradation by the proteasome [45,66,67].

**Figure 3:**
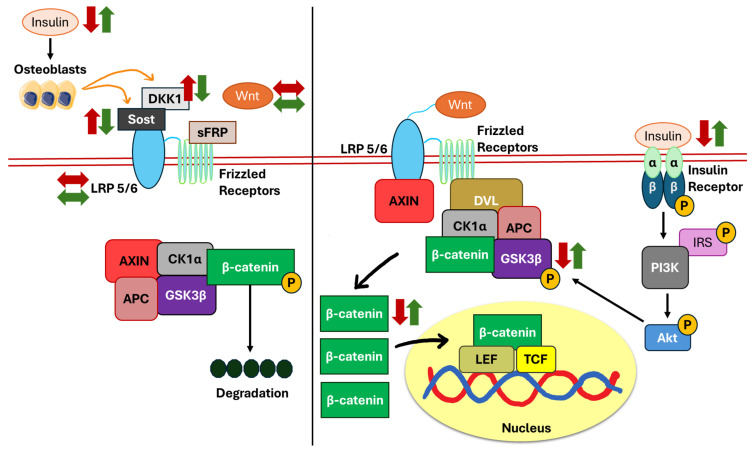
The role of the Wnt/β-catenin pathway in regulating osteogenesis and bone formation in response to insulin. Osteoblasts regulate the expression of Sost and DKK1, the Wnt inhibitors (indicated by orange arrows). The inactivation of the insulin pathway decreases phosphorylated GSK3β levels. In addition, impaired insulin signalling in diabetes increases the levels of Wnt inhibitors (Sost and DKK1) which subsequently activate GSK3β and degrade β-catenin (indicated by red arrows). The presence of insulin reinforces Wnt/β-catenin activity by reducing Sost and DKK1 expression, resulting in the phosphorylation of GSK3β and the stabilisation of β-catenin (indicated by green arrows). Abbreviations: APC, adenomatous polyposis coli; AXIN, axis inhibition protein; CK1α, casein kinase alpha; DKK1, Dickkopf-related protein 1; DVL, protein dishevelled; GSK3β, glycogen synthase kinase 3-beta; LEF, lymphoid enhancer-binding factor; LRP5, low-density lipoprotein receptor-related protein 5; sFRP, secreted Frizzled-related protein; Sost, sclerostin; TCF, T-cell-specific factor; P, phosphorylation; ↑, increase; ↓, decrease; ↔, no change.

In STZ-induced diabetic rats mimicking T1DM (whereby their insulin level is low), the expression levels of Wnt3a and LRP5 are unchanged. However, the expressions of Wnt inhibitors, Sost, and DKK1, were upregulated. The phosphorylated GSK3β and active β-catenin were downregulated in diabetic rats, resulting in reduced osteogenic gene expression [12]. Sost and DKK1 bind to the LRP5/6 receptor, preventing Wnt ligands from binding and activating the pathway. As a result, a destruction complex is formed, leading to the increased ubiquitination of β-catenin. High circulating insulin upon subcutaneous insulin infusion did not alter Wnt3a and LRP5 expressions but reduced Sost and DKK1, resulting in higher levels of GSK3β phosphorylation and active β-catenin [12]. Collectively, these observations indicated that insulin deficiency inhibited the canonical Wnt/β-catenin and downstream signalling cascades by increasing the expression of Sost and DKK1, which can be reversed by subcutaneous insulin infusion. Unphosphorylated GSK3β is also increased when insulin is deficient or resistant to its receptor, hence directly causing β-catenin to be phosphorylated (Figure 3).

### 5.3. Receptor Activator of Nuclear Factor Kappa B (RANK)/RANKL/OPG Pathway

The RANK/RANKL/OPG pathway plays a pivotal role in bone remodelling. RANKL (primarily produced by osteoblasts and osteocytes) binds to its receptor (RANK), which is expressed on the surface of osteoclast progenitors and mature osteoclasts. This interaction modulates osteoclastogenesis and promotes bone resorption [67,69]. Upon the binding of RANKL to RANK, a cascade of intracellular signalling events is triggered. Tumour necrosis factor receptor-associated factor 6 (TRAF6) is recruited and undergoes auto-ubiquitination [70,71]. This leads to the formation of a complex consisting of transforming growth factor beta-activated kinase 1 (TAK1), TAK1 binding protein 2 (TAB2), and TAK1 binding protein 3 (TAB3). The activation of this complex results in the phosphorylation of the inhibitor of nuclear factor-kappa B (IKK) complex (which includes IKKα, IKKβ, and IKKγ), thereby activating nuclear factor-kappa B (NF-κB). Once activated, NF-κB translocates into the nucleus and drives the transcription of genes essential for osteoclast differentiation, fusion, and function [71]. OPG, a decoy receptor for RANKL, serves as a key inhibitor of this pathway by binding to RANKL and preventing its interaction with RANK, thereby suppressing osteoclastogenesis and bone resorption [67,69].

**Figure 4:**
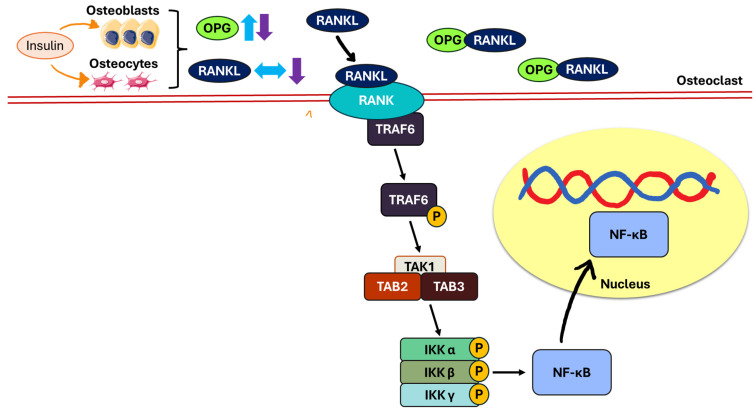
The role of the RANK/RANKL/OPG pathway in regulating osteoclastogenesis. Insulin stimulates osteoblasts and osteocytes to regulate the expression of OPG and RANKL (indicated by orange arrows). Intermittent insulin administration elevates OPG levels without altering RANKL (indicated by blue arrows), whereas continuous infusion enhances OPG and reduces RANKL expression (indicated by purple arrows) in bone tissue. Abbreviations: IKK, inhibitor of nuclear factor-kappa B; OPG, osteoprotegerin; RANK, receptor activator of nuclear factor-kappa B; RANKL, receptor activator of nuclear factor kappa-B ligand; TAK1, transforming growth factor beta-activated kinase 1; TAB2, transforming growth factor beta-activated kinase 1 binding protein 2; TAB3, transforming growth factor beta-activated kinase 1 binding protein 3; TRAF6, tumour necrosis factor receptor-associated factor 6; NF-κB, nuclear factor-kappa B; P, phosphorylation; ↑, increase; ↓, decrease; ↔, no change.

Previous studies have demonstrated that varying the frequency and duration of insulin treatment results in different outcomes in the RANK/RANKL/OPG pathway in STZ-induced animal models mimicking the pathogenesis of T1DM [25,27]. Twice-daily subcutaneous insulin injections increased OPG levels but did not change RANKL expression in STZ-induced rats after eight weeks [25]. On the other hand, T2DM rats receiving continuous subcutaneous insulin infusion for four and eight weeks had higher OPG levels and lower RANKL expressions in both their cancellous and cortical bones than T2DM rats not receiving insulin treatment [27]. These findings suggested that insulin therapy influenced bone resorption mechanisms by promoting osteoblasts and osteocytes to produce more OPG, which inhibits osteoclast activity. In addition, variations in insulin treatment regimens modulated RANKL expression differently, with continuous insulin infusion showing more favourable effects on RANKL suppression than intermittent insulin administration in bone tissues. In line with the outcomes of preclinical studies, a prospective study conducted by Basu et al. recorded an inverse relationship between OPG and insulin sensitivity in their study subjects comprising diabetic and non-diabetic individuals (*n* = 14; aged 59.9 ± 2.2 years) [62] (Figure 4).

### 5.4. Bone Morphogenic Protein-2 (BMP-2)/Suppressor of Mothers Against Decapentaplegic (Smad)-Dependent Pathway

**Figure 5:**
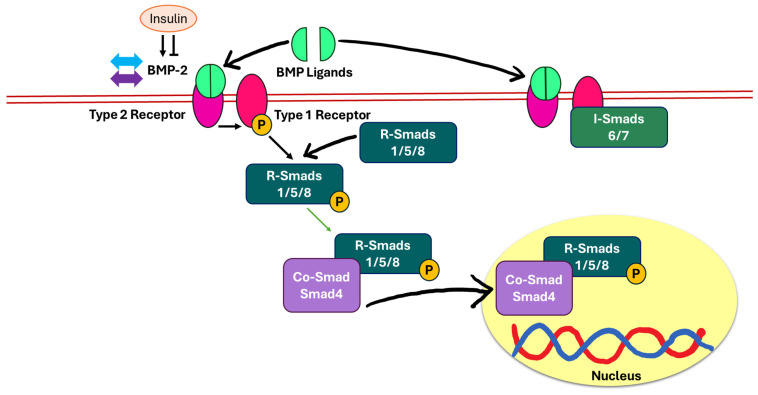
The role of the BMP-2/Smad pathway in controlling bone metabolism in response to insulin. Theoretically, insulin has both inhibitory and potentiating effects on BMP-2 signalling. In T1DM, insulin deficiency did not affect BMP-2 expression (indicated by blue arrows), despite continuous insulin infusion (indicated by purple arrows). Abbreviations: BMP, bone morphogenic protein; R-Smads, receptor-regulatory suppressors of mothers against decapentaplegic; Co-Smad, co-mediatory suppressor of mothers against decapentaplegic; I-Smads, inhibitory suppressors of mothers against decapentaplegic; P, phosphorylation; ↔, no change.

The BMP-2/Smad-dependent pathway is the key signalling mechanism in osteogenesis, chondrogenesis, and tissue repair [72,73]. BMP-2 is a member of the transforming growth factor-beta (TGF-β) superfamily [72]. The BMP-2/Smad-dependent signalling pathway is initiated when BMP ligands form a dimer and bind to a heterotetrameric complex on the cell membrane composed of type I (BMPRI) and type II (BMPRII) receptors. Following ligand binding, BMPRII activates BMPRI through phosphorylation via its serine/threonine kinase activity. This triggers the recruitment and phosphorylation of receptor-regulated Smads (R-Smads), specifically Smad1, Smad5, and Smad8. These activated R-Smads then form a complex with Smad4, also known as the common-mediator Smad (Co-Smad), and translocate into the nucleus to regulate the transcription of bone-related target genes for osteoblast differentiation and bone matrix production. This pathway is tightly regulated by inhibitory Smads (I-Smads), specifically Smad6 and Smad7. The binding of BMP-2 to BMPRI blocks R-Smad phosphorylation and activation [74]. The level of BMP-2 remained unchanged in STZ-induced diabetic rats, a model mimicking T1DM, even after insulin infusion [12]. This finding suggested that insulin may not directly stimulate BMP-2 expression in bone tissue. The responsiveness of BMP-2 expression to insulin can vary significantly between different cell types [75] (Figure 5).

## 6. Sarcopenia, Insulin Resistance, and Bone Health

Sarcopenia is a condition characterised by the progressive and generalised loss of skeletal muscle mass and strength [76]. It is increasingly recognised as a metabolic disorder with implications extending beyond impaired mobility and physical frailty. Sarcopenia predisposes individuals to insulin resistance through several key mechanisms [77]. In individuals with sarcopenia, ectopic fat accumulates within (intramyocellular) and around (intermuscular) muscle fibres. This lipid infiltration disrupts the normal insulin signalling pathways in muscle cells, compromising their glucose uptake capacity [78]. In addition, sarcopenia is marked by enhanced muscle protein degradation, which contributes to the decline in muscle mass and function. As skeletal muscle is the primary site for insulin-mediated glucose disposal, its deterioration significantly reduces insulin sensitivity, thereby increasing the risk of developing T2DM [79].

Beyond metabolic dysregulation, sarcopenia also adversely affects bone health. Sarcopenia accelerates muscle protein catabolism and reduces mechanical loading on bone, thus exacerbating bone resorption and contributing to bone loss [80]. Additionally, increased levels of myostatin (a muscle growth inhibitor) in sarcopenic individuals have been implicated in inhibiting osteoblast differentiation while promoting osteoclastogenesis [81,82]. This molecular interplay highlights the pathophysiological connection between muscle wasting and osteoporosis. Considering the increasing prevalence of sarcopenia in ageing populations and individuals with metabolic conditions, addressing its dual role in insulin resistance and skeletal deterioration is imperative. Implementing targeted interventions (such as resistance training, adequate protein intake, and the use of insulin-sensitising agents) may simultaneously support both metabolic control and musculoskeletal integrity.

## 7. Perspectives

The complex interplay between insulin and bone health has been recognised but often underexplored in the aspect of metabolic regulation. This review consolidates the findings from the current literature to elucidate the relationship between bone metabolism and insulin level, insulin resistance, insulin treatment, and insulin receptor silencing across various physiological and pathological contexts. The nature of insulin deficiency, whether absolute (the pancreas produces very little or no insulin) or relative (insulin is insufficient to overcome insulin resistance), distinctly influences bone microarchitecture and strength [12,14,16,19]. Insulin is an anabolic hormone responsible for bone formation in trabecular and cortical regions. In T1DM, absolute insulin deficiency leads to reduced osteoblast activity and impaired bone formation, adversely affecting both trabecular and cortical bone [13]. T2DM is associated with hyperinsulinaemia, indicating that insulin signalling may be preserved in bone. As a result, the dense cortical bone undergoes less active remodelling compared to the metabolically dynamic trabecular bone with a honeycomb-like structure [83]. Variations in frequency, duration, and route of insulin administration significantly impact bone health [12,25,27], highlighting the importance of optimising insulin regimens for skeletal benefits. To maximise its positive effects, endogenous (produced by the body) or exogenous (externally supplied) insulin exposure should be sustained over time for better outcomes. The insulin receptor silencing studies provide insights into the critical role of insulin signalling in the expression of osteoprogenitors and osteoblasts for bone development and maintenance. Thus, insulin receptors may serve as a promising therapeutic target for bone metabolic disorders, particularly in addressing diabetes-related skeletal complications.

Human studies present a more complex narrative on the relationship between insulin and bone health due to the physiological variability across age, gender, body weight, and disease status. Insulin resistance is predominantly linked to compromised bone integrity. However, hyperinsulinaemia and insulin resistance may support bone health in younger adults and postmenopausal women, likely due to confounding factors such as the increased mechanical loading associated with higher body weight in these individuals. These inconsistencies highlight the urgent need for well-designed longitudinal studies to evaluate causal relationships and distinguish the direct effects of insulin and insulin resistance from other metabolic influences on bone health. The clinical utility in preventing or mitigating diabetic bone disease remains uncertain. Variations in diabetes progression, glycaemic control, and patient demographics may influence the efficacy of insulin treatment on bone outcomes. In addition, it appears that oral glucose-lowering medications work synergistically with insulin to enhance bone health [57,58]. Future research should also incorporate comprehensive skeletal assessments, including bone turnover markers, fracture risk, and microarchitectural analysis.

The optimisation of insulin regimen in diabetic patients who are at risk of bone fragility warrants careful consideration. The mode of insulin delivery may influence their effects. Continuous subcutaneous insulin infusion (also known as insulin pump therapy) provides more stable glycaemic control with reduced glycaemic variability compared to multiple daily injections or intermittent bolus administration. Evidence from preclinical studies suggested that continuous subcutaneous insulin infusion may exert more favourable effects on bone turnover markers and BMD, potentially due to enhanced insulin sensitivity and a reduction in glycaemic fluctuations, which are known to negatively affect the bone remodelling process.

The co-administration of insulin and anti-osteoporotic therapies (such as bisphosphonates) presents both clinical opportunities and challenges. Bisphosphonates are potent antiresorptive agents widely used to treat osteoporosis. Increasing attention has been directed towards the pleiotropic effects of bisphosphonates, including their anti-hyperglycaemic properties. In a randomised controlled trial, the weekly oral administration of alendronate (70 mg) was shown to reduce fasting blood glucose levels, HbA1c levels, and insulin resistance in postmenopausal women [84]. In an earlier study, Maugeri et al. reported that alendronate treatment not only improved BMD but also decreased daily insulin requirements in patients with insulin-dependent senile diabetes and osteoporosis [85]. Although bisphosphonates are generally considered safe for individuals with normal renal function, the use of bisphosphonates may cause a decline in glomerular filtration rate in patients with pre-existing kidney impairment or when administered at high doses. Considering that diabetes is a leading cause of chronic kidney disease, caution is warranted when prescribing bisphosphonates in this patient papulation. Careful assessment of renal function and individualised dosing strategies are essential to minimise potential nephrotoxic risks.

Insulin signalling is pivotal for maintaining bone homeostasis. The disruption of insulin signalling (due to insulin deficiency or resistance) results in significant skeletal complications via several tightly coordinated key pathways, including PI3K/Akt, canonical Wnt/β-catenin, and RANK/RANKL/OPG signal transduction cascades. These pathways are not isolated and exhibit extensive crosstalk with signalling components from each network influencing and integrating with one another to regulate bone metabolism. A severe reduction in insulin leads to an overall suppression of the PI3K/Akt pathway via the phosphorylation of Akt and the downstream activation of GSK3β. This results in decreased osteoblast survival, reduced expression of the osteogenic transcription factor Runx2, and impaired glucose uptake, which collectively compromise matrix production and bone formation [86]. The downstream effects of insulin-induced PI3K/Akt activation includes the upregulation of OPG expression and the downregulation of RANKL expression, thereby reducing osteoclastogenesis and favouring bone formation [87]. Simultaneously, PI3K/Akt signalling intersects with the canonical Wnt/β-catenin pathway. In Wnt signalling, a lack of insulin increases the expression of Wnt inhibitors (Sost and DKK1), allowing GSK3β to remain active, leading to the degradation of β-catenin. The downregulation of β-catenin impairs osteoblast differentiation and shifts mesenchymal stem cells toward adipogenic lineages, increasing marrow fat and weakening bone [88]. GSK3β has emerged as a common regulatory molecule connecting the insulin-regulated PI3K/Akt pathway and bone-related Wnt/β-catenin pathway [37]. Moreover, the Wnt/β-catenin signalling modulates the OPG/RANKL ratio. The activation of β-catenin favours OPG expression while repressing RANKL, exerting anti-resorptive effects. The insulin-deficient state also disrupts the balance of the RANK/RANKL/OPG system by reducing OPG, thereby promoting osteoclastogenesis and enhancing bone resorption [69]. The convergence of PI3K/Akt and Wnt/β-catenin signalling highlights their potential as therapeutic targets. Stimulating these pathways not only enhance bone formation but also suppress osteoclast-mediated resorption through upregulation of OPG and inhibition of RANKL. Hence, the modulation of these interconnected pathways using pharmacological agents (such as metformin, Wnt activators, or GSK3β inhibitors) offers promising potential in the management of both diabetes and osteoporosis.

Several limitations that hinder a comprehensive understanding of the relationship between insulin and bone health must be acknowledged. Firstly, the predominance of cross-sectional design in human studies limits the ability to establish causality and temporal associations between insulin dysregulation and bone deterioration. Secondly, the variability in age, gender, ethnicity, menopausal status, and comorbid conditions further complicates the ability to generalise findings across diverse demographic groups. Thirdly, the inconsistencies in assessing insulin resistance (including the use of HOMA-IR, QUICKI, and TyG) as well as bone health (including bone densitometry, turnover markers, microarchitecture, and strength) contribute to the variability and divergence in findings across studies. Fourthly, findings on the effects of insulin on bone health remain ambiguous, with outcomes influenced by disease stage, glycaemic control, treatment duration, and concurrent medications. Despite the well-established research on the association between skeletal health and circulating insulin level, insulin resistance, and insulin therapy, the underlying signalling pathways mediating insulin’s action in bone cells are not well defined.

Several future directions are recommended to deepen our understanding of how insulin influences bone metabolism. Longitudinal prospective cohorts that track insulin levels, insulin resistance, and bone health over time are essential to clarify the causal and temporal relationships. Standardising the indices used to assess insulin resistance and skeletal parameters should be adopted to improve comparability across studies. Given the roles of sex hormones and ageing in orchestrating both insulin action and bone metabolism, age- and gender-specific analyses should be incorporated in both preclinical and clinical research. In addition, human studies should ensure the inclusion of diverse populations to uncover potential ethnic or genetic predispositions influencing the insulin–bone relationship. Future clinical trials should consider incorporating bone-related endpoints to evaluate the efficacy of insulin and insulin-sensitising agents (such as metformin and glucagon-like peptide-1) on skeletal outcomes, particularly in diabetic populations. Leveraging multi-omics approaches can uncover novel biomarkers and molecular pathways linking insulin metabolism to bone physiology. Of particular interest, GSK3β (a point of convergence in multiple key signalling pathways) presents a promising drug target for the diagnosis, prognosis, management, and treatment monitoring of metabolic bone diseases.

**Figure 6:**
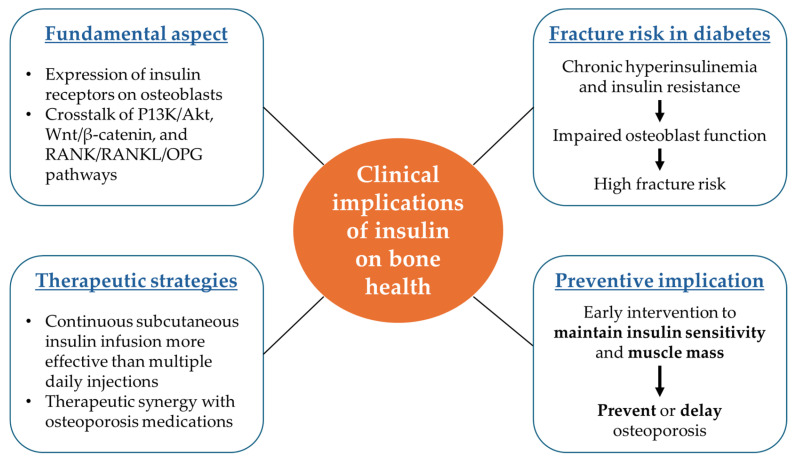
Clinical implications of insulin on bone health.

By addressing current limitations and pursuing targeted research strategies, a clearer understanding of the insulin–bone axis can be achieved, ultimately driving the development of personalised interventions to preserve bone health in individuals with metabolic disorders. The clinical implications of insulin on bone health have been summarised (Figure 6).

## 8. Conclusions

Insulin emerges as a key modulator of bone health, functioning as a systemic hormone and a local regulator of bone cell activity. The relationship between insulin and the skeletal system remains complex and partially understood, with inconsistencies across studies driven by differences in experimental models, patient demographics, and assessment methods. It is evident that the effects of insulin on bone are highly context-dependent, influenced by age, gender, hormonal status, and metabolic environment. Notably, skeletal manifestations vary between T1DM and T2DM, reflecting the distinct pathophysiological mechanisms of insulin deficiency and resistance. A deeper mechanistic insight into the action of insulin on bone, supported by robust longitudinal human studies and standardised evaluation approaches, is crucial to unravelling this intricate relationship. Advancing the understanding of the insulin–bone axis may open new avenues for novel targeted strategies to predict, prevent, diagnose, and manage skeletal complications in populations at risk of both metabolic and skeletal disorders.

## Figures and Tables

**Table 2:** The relationship between insulin treatment on bone health in animal studies.

Type of Animal Model	Treatment/Intervention (Dose, Route and Duration)	Findings	Reference
STZ-induced diabetic rats	Neutral protamine Hagedorn insulin (2.5 IU twice daily, subcutaneous injection, 8 weeks)	Calcium: ↑; ionised calcium: ↔; phosphorus: ↔; ALP: ↓; Tb.Sp: ↓; trabecular width: ↑; trabecular bone area: ↑; load: ↔; stiffness: ↔; stress: ↔; strain: ↔; Young’s modulus: ↑; RANKL: ↔; OPG: ↑; OCN: ↑	[25]
STZ induced diabetic rats	Neutral protamine Hagedorn (100 IU twice daily, subcutaneous injection, 30 days)	Glucose: ↓; alveolar bone loss: ↓; OPG: ↑; RANKL: ↔	[26]
STZ-induced diabetic mice	Humulin R insulin (0.25 IU/day, osmotic minipump, 4 weeks)	Glucose: ↓; CTX: ↓; P1NP: ↑; stiffness: ↑; peak force: ↑; femoral length: ↑; modulus: ↔; bending strength: ↔; toughness: ↔ Cortical parameters: Ct.Ar: ↑; Ct.Th: ↑; mineral density: ↔ Trabecular parameters: BV/TV: ↑; Tb.N: ↑; Tb.Th: ↑; mineral density: ↑	[13]
STZ-induced diabetic rats	Insulin from bovine pancreas (1.6 IU/day, continuous subcutaneous infusion, 4 weeks)	Insulin: ↑; insulin receptor: ↔; femur length: ↑; tibia length: ↑; bone weight (femur, tibia, proximal tibia): ↑; ALP: ↑; TRAP: ↔; CTSK: ↔; calcium: ↑; hydroxyproline: ↑; deoxypyridinoline: ↔; BV/TV: ↑; trabecular bone surface: ↑; Tb.Th: ↑; Tb.N: ↑; Ob.N: ↑; Oc.N: ↔; BMP-2: ↔; Dlx5: ↑; Runx2: ↑; OSX: ↑; OCN: ↑; COL1: ↑; Wnt3a: ↔; LRP5: ↔; Akt: ↔; p-Akt: ↑; GSK3β: ↔; p-GSK3β: ↑; β-catenin: ↑; p-β-catenin: ↑; Sost: ↓; DKK1: ↓	[12]
Zucker diabetic fatty rats fed high-fat, high-carbohydrate diet	Insulin glargine (0.5–13.0 IU/ daily, subcutaneous injection, 12 weeks)	Non-enzymatic glycation: ↔; tissue mineral density ratio: ↔; elastic modulus: ↔; toughness: ↔; stress: ↔ Cortical parameters: Porosity: ↔	[20]
Zucker diabetic fatty rats with subcritical femur defects	Insulin glargine (0.5–13.0 IU/ daily, subcutaneous injection, 12 weeks)	Glucose: ↓; HbA1c: ↓; calcium: ↔; phosphate: ↔; P1NP: ↑; CTX: ↔; TRAP: ↑; PTH: ↑; force: ↔; work to failure: ↔; BFR: ↑ Cortical parameters: BV/TV: ↑; BMD: ↔; Ct.Th: ↔ Trabecular parameters: BV/TV: ↔; BMD: ↔; Tb.N: ↔; Tb.Th: ↔	[21]
STZ- and high-fat-diet-induced diabetic rats	Insulin (2.5 IU/day, continuous subcutaneous infusion, 4 weeks)	Glucose: ↓; OCN: ↑; TRAP: ↔; CTX: ↓; BMD: ↔; load: ↔; elasticity: ↔; toughness: ↑; matrix mineralisation: ↓ Trabecular parameters: BV/TV: ↑; Tb.N: ↑; Tb.Th: ↑; Tb.Sp: ↓; indentation modulus: ↔; hardness: ↔; surface grain size: ↔; roughness: ↔; OPG: ↑; RANKL: ↓ Cortical parameters: Indentation modulus: ↔; hardness: ↔; surface grain size: ↔; roughness: ↓; OPG: ↑; RANKL: ↓	[27]
	Insulin (2.5 IU/day, continuous subcutaneous infusion, 8 weeks)	Glucose: ↔; OCN: ↑; TRAP: ↓; CTX: ↓; BMD: ↔; load: ↑; elasticity: ↑; toughness: ↑; matrix mineralisation: ↓ Trabecular parameters: BV/TV: ↑; Tb.N: ↑; Tb.Th: ↑; Tb.Sp: ↓; indentation modulus: ↔; hardness: ↑; surface grain size: ↓; roughness: ↔; OPG: ↑; RANKL: ↓ Cortical parameters: Indentation modulus: ↑; hardness: ↑; surface grain size: ↓; roughness: ↓; OPG: ↑; RANKL: ↓	

Abbreviations: Akt, protein kinase B; ALP, alkaline phosphatase; BFR, bone formation rate; BMD, bone mineral density; BMP-2, bone morphogenetic protein-2; BV/TV, bone volume/total volume; COL1, type 1 collagen; Ct.Ar, cortical area; Ct.Th, cortical thickness; CTSK, cathepsin K; CTX, carboxyl-terminal cross-linked telopeptide of type 1 collagen; DKK1, Dickkopf-related protein 1; Dlx5, distal-less homeobox 5; GSK3β, glycogen synthase kinase-3 beta; HbA1c, glycated haemoglobin A1c; PTH, parathyroid hormone; LRP5, low-density lipoprotein receptor-related protein 5; Ob.N, osteoblast number; OCN, osteocalcin; Oc.N, osteoclast number; OPG, osteoprogeterin; OSX, osterix; P1NP, N-terminal propeptide of type 1 collagen; p-Akt, phosphorylated protein kinase B; p-β-catenin, phosphorylated beta-catenin; p-GSK3β, phosphorylated glycogen synthase kinase-3 beta; RANKL, receptor activator of nuclear factor kappa-B ligand; Runx2, runt-related transcription factor 2; Sost, sclerostin; Tb.N, trabecular number; Tb.Sp, trabecular separation; Tb.Th, trabecular thickness; TRAP, tartrate-resistant acid phosphatase; STZ, streptozotocin; Wnt3a, Wnt ligand 3a; β-catenin, beta-catenin; ↑, increase; ↓, decrease; ↔, no change.

**Table 3:** Effects of insulin receptor silencing on bone health in animal studies.

Type of Animal Model	Findings	Reference
Mice with IRβ deletion (15 weeks)	Cortical parameters: BV: ↔; BV/TV: ↔; Ct.Th: ↔; mineral density: ↔ Trabecular parameters: BV/TV: ↔; Tb.N: ↔; Tb.Sp: ↔; Tb.Th: ↔; ultimate torque: ↔; torsional rigidity: ↔	[28]
Mice with IRβ deletion (48 weeks)	Cortical parameters: BV: ↔; BV/TV: ↔; Ct.Th: ↔; mineral density: ↔ Trabecular parameters: BV/TV: ↓; Tb.N: ↔; Tb.Sp: ↔; Tb.Th: ↔; ultimate torque: ↔; torsional rigidity: ↓	
Mice lacking insulin receptors in osteoblasts	Insulin: ↓; glucose: ↑; BV/TV: ↓; Tb.N: ↓; Tb.Th: ↓; Tb.Sp: ↑; Ob.N: ↓; BFR: ↔; Oc.N: ↔; erosion depth: ↓; OCN: ↓; CTX: ↓; OPG: ↑	[29]
Female mice with osteoprogenitor-selective ablation of insulin receptors	Femur length: ↓; femur slenderness: ↑; force: ↓; stiffness: ↓; elasticity: ↔; binding strength: ↔; OCN: ↔; CTX: ↔ Cortical parameters: Ct.Th: ↓; Ct.Ar: ↓; mineral density: ↓ Trabecular parameters: BV/TV: ↔; Tb.Th: ↔; Tb.N: ↔; Tb.Sp: ↔; SMI: ↓; mineral density: ↔	[30]
Male mice with osteoprogenitor-selective ablation of insulin receptors	Femur length: ↓; femur slenderness: ↑; force: ↓; stiffness: ↔; elasticity: ↔; binding strength: ↔; OCN: ↔; CTX: ↓ Cortical parameters: Ct.Th: ↓; Ct.Ar: ↓; mineral density: ↓ Trabecular parameters: BV/TV: ↓; Tb.Th: ↓; Tb.N: ↔; Tb.Sp: ↔; SMI: ↑; mineral density: ↔; binding strength: ↔; OCN: ↔; CTX: ↑	
Transgenic insulin receptor knockout mice	Tibial length: ↔; pyridinoline: ↔; Runx2: ↔; OCN: ↔ Cortical parameters: BMC: ↔; BMD: ↔; volume fraction: ↔; Ct.Th: ↔; inner perimeter: ↓; medullary area: ↓; BA/TA: ↑ Trabecular parameters: BMC: ↔; BMD: ↔; volume fraction: ↔; Tb.Th: ↔; Tb.Sp: ↔; Conn.D: ↔	[31]

Abbreviation: BA/TA, bone area/volume area; BFR, bone formation rate; BMC, bone mineral content; BMD, bone mineral density; BV, bone volume; BV/TV, bone volume/total volume; Conn.D, connectivity density; Ct.Ar, cortical area; Ct.Th, cortical thickness; CTX, collagen type 1 cross-linked carboxyl-terminal telopeptide; IRβ, insulin receptor beta; Ob.N, osteoblast number; Oc.N, osteoclast number; OCN, osteocalcin; OPG, osteoprotegerin; SMI, structural model index; Tb.N, trabecular number; Tb.Sp, trabecular spacing; Tb.Th, trabecular thickness; Runx2, runt-related transcription factor 2; ↑, increase; ↓, decrease; ↔, no change.

**Table 4:** Relationship between insulin levels and bone health in human studies.

Type of Study	Subject Characteristics	Findings	References
Cross-sectional study	Non-diabetic postmenopausal women with suspected or diagnosed osteoporosis (*n* = 381; aged 62 ± 9 years old)	Women with higher insulin level had higher hip BMD and volumetric BMD.	[32]
Cross-sectional study	Non-diabetic postmenopausal women (*n* = 437; aged 51–56 years)	Fasting insulin level was positively associated with BMD (β = 0.033).	[33]
Cross-sectional study	Boys (*n* = 1344; age = 15.47 ± 0.3 years old) and girls (*n* = 1440; aged 15.48 ± 0.3 years old)	Insulin level was negatively correlated with cortical BMD and periosteal circumference.	[34]
Cross-sectional study	Participants in biomarker project of Midlife in the United States Study (*n* = 717; aged 56.8 ± 11.3 years old)	A higher level of fasting insulin was associated with lower femoral neck (effect size = −0.099; 95% CI −0.19, −0.01) and lumbar spine BMD (effect size = −0.129; 95% CI −0.23, −0.03). A higher level of fasting insulin was associated with lower compression (effect size = −0.121; 95% CI −0.19, −0.05), bending (effect size = −0.180; 95% CI −0.27, −0.09), and impact (effect size = −0.158; 95% CI −0.25, −0.07) strength.	[35]
Cross-sectional study	Young men (*n* = 113; aged 19.8 ± 2.3 years old) and women (*n* = 353; aged 19.4 ± 2.2 years old)	Insulin level was positively associated with total body BMC, mediated by lean mass.	[36]

Abbreviations: BMC, bone mineral content; BMD, bone mineral density; CI, confidence interval.

**Table 5:** Relationship between insulin resistance and bone health in humans.

Type of Study	Subject Characteristics	Findings	Reference
Cross-sectional study	Iranian boys and girls (*n* = 423; aged 13.93 ± 2.64 years old)	HOMA-IR was correlated with low lumbar spine BMC (β = −1.1; *p* = 0.008), BMD (β = −0.01; *p* = 0.011), BMAD (β = −0.002; *p* = 0.029), and z-score (β = −0.105; *p* = 0.009); low femoral neck BMC (β = −0.06; *p* = 0.004) and BMD (β = −0.010; *p* = 0.010); and low whole-body BMD (β = −0.005; *p* = 0.029) and z-score (β = −0.076; 0.036). QUICKI was associated high lumbar spine BMC (β = 37.21; *p* = 0.0001), BMD (β = 0.277; *p* = 0.007), BMAD (β = 0.062; *p* = 0.026), and z-score (β = 2.63; *p* = 0.009); and high femoral neck BMC (β = 1.297; *p* = 0.013).	[44]
Cross-sectional study	Participants in the NHANES (*n* = 5292; aged ≥ 18 years old)	Subjects with HOMA-IR ≥ 2 and HOMA-β < 100 had a higher risk of osteoporosis (OR = 1.070; 95% CI 0.656, 1.744) as compared to subjects with HOMA-IR < 2 and HOMA-β < 100. Subjects with HOMA-IR ≥ 2 and HOMA-β ≥ 100 had a higher risk of osteoporosis (OR = 1.256; 95% CI 0.625, 2.526) as compared to subjects with HOMA-IR < 2 and HOMA-β < 100.	[45]
Longitudinal study	Chinese adults without low bone mass or osteoporosis (*n* = 8770; aged ≥ 18 years old)	TyG index was negatively associated with bone mass (HR = 1.56; 95% CI: 1.25, 1.93), osteoporosis (HR = 1.66; 95% CI: 1.06, 2.59), and both (HR = 1.55; 95% CI: 1.27, 1.88).	[46]
Cross-sectional study	Participants in biomarker project of Midlife in the United States Study (*n* = 717; aged 56.8 ± 11.3 years old)	HOMA-IR was negatively associated with femoral neck compression (effect size = −0.091; 95% CI: −0.153, −0.030), bending (effect size = −0.141; 95% CI: −0.222, −0.060), and impact (effect size = −0.141; 95% CI: −0.222, −0.048) strength. HOMA-IR was negatively associated with lumbar spine BMD (effect size = −0.087; 95% CI: −0.171, −0.002).	[35]
Cross-sectional study	Men and women (*n* = 525; aged ≥ 60 years old)	Insulin resistance was associated with low CTX (estimate = −24.8%; 95% CI: −38.9, −7.5) in men. T2DM was associated with low bone turnover marker levels in men [CTX (estimate = −34.7%; 95% CI: −48.1, −17.8)] and women [OCN (estimate = −31.9%; 95% CI: −41.8, −20.4), P1NP (estimate = −26.0%; 95% CI: −38.7, −10.8), and CTX (estimate = −30.9%; 95% CI: −46.2, −11.3)].	[47]
Cross-sectional study	Subjects with dysglycaemia (*n* = 5277; aged ≥ 18 years old)	HOMA-IR was negatively associated with CTX (β = −0.044; 95% CI −0.053, −0.035), P1NP (β = −7.340; 95% CI −9.130, −5.550), and OCN (β = −2.885; 95% CI −3.357, −2.412). HOMA-β was positively associated with CTX (β = 0.022; 95% CI 0.014, 0.031), P1NP (β = 6.951; 95% CI 5.300, 8.602), and OCN (β = 1.361; 95% CI 0.921, 1.800).	[48]
Cohort study	Premenopausal (*n* = 861; aged 45.44 ± 2.51 years old), menopausal transition (*n* = 571; aged 50.71 ± 2.48 years old), and postmenopausal (*n* = 693; aged 55.11 ± 3.35 years old) women	Slower BMD loss was observed when HOMA-IR level was <2.82, while faster BMD loss was evident when HOMA-IR level was ≥2.82 in all groups.	[49]
Cross-sectional study	Non-diabetic postmenopausal women (*n* = 1008; aged ≥ 50 years old)	HOMA-IR was associated with low total bone volume at the femoral neck (β = −0.12), intertrochanter (β = −0.43), and total proximal femur (β = −0.76); low cortical volume at the femoral neck (β = −0.05); high cortical BMD at the femoral neck (β = 23.0), intertrochanter (β = 6.8), and total proximal femur (β = 12.2); and low femoral neck strength indices [estimated cortical depth (β = −0.011)], compressive strength index (β = −0.013), section modulus (β = −0.017)	[50]
Cross-sectional study	Overweight and obese men and women (*n* = 79; aged 62.8 ± 7.9 years old)	HOMA-IR was associated with low proximal radius cortical BMD in women (β = −4.79; 95% CI −8.66, −0.92). There was no association between HOMA-IR and bone parameters in men.	[51]
Cross-sectional study	T2DM patients (*n* = 234; aged 57.5 ± 10.8 years old)	HOMA-IR was associated with higher risk of osteoporosis in females (OR = 2.63; 95% CI 1.15, 5.99).	[52]
Cross-sectional study	Men and women from United States (*n* = 5456; aged 30.33 ± 13.55 years old)	TyG (β = 0.0124; 95% CI 0.0006, 0.0242), TyG-WC (β = 0.0001; 95% CI 0.0001, 0.0001), TyG-WHtR (β = 0.0116; 95% CI 0.0076, 0.0156), and TyG-BMI (β = 0.0004; 95% CI 0.0003, 0.0004) were associated with high total BMD.	[53]
Cohort study	Postmenopausal women (*n* = 81; aged 58.40 ± 6.08 years old)	Higher left femur and total hip BMD was observed in the insulin resistant than the non-insulin resistant group.	[54]
Cross-sectional study	Non-diabetic postmenopausal women (*n* = 437; aged 51–56 years old)	HOMA-IR (β = 0.139), HOMA-β (β = 0.137), and MET-IR (β = 0.145) were positively associated with femoral neck BMD. HOMA-IR (β = 0.131), HOMA-β (β = 0.134), and MET-IR (β = 0.138) were positively associated with femoral neck T-score.	[33]
Cross-sectional study	Non-diabetic postmenopausal women with suspected or diagnosed osteoporosis (*n* = 381; mean aged 62 ± 9 years old)	Women with higher HOMA-IR values had higher hip BMD and volumetric BMD. No association was found between HOMA-IR and trabecular bone score.	[32]
Retrospective study	Postmenopausal T2DM patients with (*n* = 91; aged 68.74 ± 7.89 years old) and without osteoporosis (*n* = 119; aged 61.94 ± 7.33 years old)	METS-IR was positively associated with lumbar spine (β = 0.006), femoral neck (β = 0.005), and hip (β = 0.005) BMD.	[55]

Abbreviation: BMAD, bone mineral apparent density; BMC, bone mineral content; BMD, bone mineral density; BMI, body mass index; CI, confidence interval; CTX, collagen type 1 cross-linked carboxyl-terminal telopeptide; HOMA-IR, homeostatic model assessment of insulin resistance; HOMA-β, homeostatic model assessment of β-cell function; HR, hazard ratio; MET-IR, metabolic score for insulin resistance; NHANES, National Health and Nutrition Examination Survey; OCN, osteocalcin; OR, odds ratio; P1NP, procollagen type 1 N-terminal propeptide; QUICKI, quantitative insulin sensitivity check index; TyG index, triglyceride–glucose index; TyG-BMI, TyG index with body mass index; TyG-WC, TyG index with waist circumference; TyG-WHtR, TyG index with waist-to-height ratio; T2DM, type 2 diabetes mellitus.

**Table 6:** Relationship between insulin treatment and bone health in human studies.

Type of Study	Subject Characteristics	Findings	Reference
Cohort study	Healthy postmenopausal women, postmenopausal women with osteoporosis, T2DM postmenopausal women with insulin treatment once daily, and T2DM postmenopausal women with metformin (500 mg) treatment twice daily (*n* = 200; aged 50–73 years old)	The levels of BMD were higher and OCN and CTX were lower in T2DM women taking insulin compared to postmenopausal women with osteoporosis.	[57]
Retrospective study	Non-diabetic controls (*n* = 30; aged 42.34 ± 6.71 years old), T2DM subjects receiving oral glucose-lowering medication (*n* = 25; aged 44.44 ± 8.13 years old), and T2DM subjects receiving oral glucose-lowering medication with insulin glargine injection (*n* = 25; aged 42.60 ± 9.25 years old)	T2DM subjects receiving oral glucose-lowering medication with insulin glargine injection had higher lumbar vertebrae and spine BMD and serum calcium levels but lower serum phosphate levels as compared to those taking oral glucose-lowering medication only.	[58]
Cohort study	Cohort 1: Healthy adults (*n* = 8; age: 37–63 years)	CTX: ↓; TRAP: ↔; ucOCN: ↔; OCN: ↔; P1NP: ↔.	[59]
	Cohort 2: Healthy young men (*n* = 12; age = 18–34 years)	CTX: ↓; TRAP: ↔; ucOCN: ↓; OCN: ↔; P1NP: ↔.	
	Cohort 3: Healthy elderly women (*n* = 13; age = 69–79 years)	CTX: ↔; TRAP: ↓; ucOCN: ↓; OCN: ↓; P1NP: ↔.	
Cohort study	Women with T2DM using insulin (*n* = 55; aged 53.9 ± 5.7 years old) and not using insulin (*n* = 55; aged 53.3 ± 4.9 years old)	Insulin users experienced a greater loss in BMD at the femoral neck than non-insulin users, while BMD at spine and total hip were not affected.	[60]
Cohort study	T2DM patients using insulin (*n* = 2979; aged 61.7 ± 11.9 years old) and not using insulin (*n* = 14,895; aged 61.8 ± 11.9 years old)	Major fracture rates were higher in insulin users than non-insulin users. An association was confirmed between insulin use and fracture risk (subhazard ratio = 1.38; 95% CI 1.06, 1.80)	[61]
Cohort study	Diabetic (*n* = 7; aged 62.0 ± 1.8 years old) and non-diabetic (*n* = 7; aged 57.9 ± 4.0 years old) subjects	P1NP, OCN, ucOCN, CTX, and OPG levels were not significantly different at the end of low-, intermediate- and high-dose insulin treatment.	[62]

Abbreviations: BMD, bone mineral density; CI, confidence interval; CTX, collagen type 1 cross-linked carboxyl-terminal telopeptide; OCN, osteocalcin; P1NP, procollagen type 1 N-terminal propeptide; TRAP, tartrate-resistant acid phosphatase; T2DM, type 2 diabetes mellitus; ucOCN, undercarboxylated osteocalcin; ↓, decrease; ↔, no change.

## Data Availability

Not applicable.

## Abbreviations

The following abbreviations are used in this manuscript:

Akt	Protein kinase B
ALP	Alkaline phosphatase
APC	Adenomatous polyposis coli
AXIN	Axis inhibition protein
BA/TA	Bone area/total area
BFR	Bone formation rate
BMAD	Bone mineral apparent density
BMC	Bone mineral content
BMD	Bone mineral density
BMI	Body mass index
BMP-2	Bone morphogenic protein-2
BMPRI	BMP type I receptor
BMPRII	BMP type II receptor
BSP	Bone sialoprotein
BV/TV	Bone volume/total volume
CI	Confidence interval
CK1α	Casein kinase alpha
COL1	Type I collagen
Conn.D	Connectivity density
Co-Smad	Common-mediator Smad
CTSK	Cathepsin K
Ct.Ar	Cortical area
Ct.Ar/Tt.Ar	Cortical area fraction
Ct.Th	Cortical thickness
CTX	Carboxyl-terminal cross-linked telopeptide of type 1 collagen
DKK1	Dickkopf-related protein 1
dLS	Double-labelled surface
Dlx5	Distal-less homeobox 5
DVL	Protein dishevelled
ES	Eroded surface
Foxo1	Forkhead box protein O1
GLUT4	Glucose transporter 4
GSK3β	Glycogen synthase kinase 3-beta
HOMA-IR	Homeostatic model assessment of insulin resistance
HOMA-β	Homeostatic model assessment of β-cell function
HR	Hazard ratio
IKK	Inhibitor of nuclear factor-kappa B
IRS	Insulin receptor substrate
IRβ	Insulin receptor β
I-Smads	Inhibitory Smads
KK-Ay	Yellow Kuo Kondo
LEF	Lymphoid enhancer-binding factor
LRP5	Low-density lipoprotein receptor-related protein 5
MAR	Mineral apposition rate
METS-IR	Metabolic score for insulin resistance
MS	Mineralising surface
NF-κB	Nuclear factor-kappa B
NHANES	National Health and Nutrition Examination Survey
Ob.N	Osteoblast number
Ob.S	Osteoblast surface
Oc.N	Osteoclast number
Oc.S	Osteoclast surface
OCN	Osteocalcin
OPG	Osteoprotegerin
OR	Odds ratio
OS	Osteoid surface
OSX	Osterix
OV	Osteoid volume
PIP_2_	Phosphorylates phosphatidylinositol 4,5-bisphosphate
PIP_3_	Phosphatidylinositol 3,4,5-trisphosphate
PI3K	Phosphatidylinositol 3-kinase
P1NP	N-terminal propeptide of type 1 collagen
p-Akt	Phosphorylated protein kinase B
p-β-catenin	Phosphorylated β-catenin
p-GSK3β	Phosphorylated glycogen synthase kinase 3-beta
QUICKI	Quantitative insulin sensitivity check index
RANK	Receptor activator of nuclear factor kappa B
RANKL	Receptor activator of nuclear factor kappa-B ligand
R-Smads	Receptor-regulated Smads
Runx2	Runt-related transcription factor 2
sFRP	Secreted Frizzled-related proteins
sLS	Single-labelled surface
Smad	Suppressor of mothers against decapentaplegic
SMI	Structure model index
Sost	Sclerostin
STZ	Streptozotocin
TAB2	TAK1 binding protein 2
TAB3	TAK1 binding protein 3
TAK1	Transforming growth factor beta-activated kinase 1
Tb.N	Trabecular number
Tb.Sp	Trabecular separation
Tb.Th	Trabecular thickness
TCF	T-cell-specific factor
TGF-β	Transforming growth factor-beta
TRAF6	Tumour necrosis factor receptor-associated factor 6
TRAP	Tartrate-resistant acid phosphatase
Tt.Ar	Total cross-sectional area inside the periosteal envelope
TyG	Triglyceride–glucose index
TyG-BMI	Tyg index with body mass index
TyG-WC	Tyg index with waist circumference
TyG-WHtR	Tyg index with waist-to-height ratio
T1DM	Type 1 diabetes mellitus
T2DM	Type 2 diabetes mellitus
ucOCN	Uncarboxylated osteocalcin
V-ATPase	V-type proton atpase
Wnt3a	Wnt ligand 3a
β-catenin	Beta-catenin
